# 

**Published:** 1909-05

**Authors:** 


					﻿Sixty=fourth Year.
Vol. LXIV.
MAY, 1909.
No. io
BUFFALO
ItyOW&UJOURNAL
Established 184S by AUSTIN FLINT M. D.
X, O. VI. VZ.
EDITOR:
WILLIAM WARREN POTTER, M. D.
ASSISTANT EDITORS:
WILLIAM C. KRAUSS, M. D.	NELSON W. WILSON, M. D
ASSOCIATE EDITORS:
JAMES WRIGHT PUTNAM, M. D. ERNEST WENDE, M.D.
JOHN PARMENTER, M. D.	fOHN A. MILLER, Ph. D.
MAUD J. FRYE, M. D.
				

